# Clinical Characteristics of Pediatric Patients With Sellar and Suprasellar Lesions Who Initially Present With Central Diabetes Insipidus: A Retrospective Study of 55 Cases From a Large Pituitary Center in China

**DOI:** 10.3389/fendo.2020.00076

**Published:** 2020-02-20

**Authors:** Xin Ji, Zihao Wang, Wenze Wang, Lu Gao, Xiaopeng Guo, Chenzhe Feng, Wei Lian, Kan Deng, Bing Xing

**Affiliations:** ^1^Department of Neurosurgery, Chinese Academy of Medical Sciences and Peking Union Medical College, Peking Union Medical College Hospital, Beijing, China; ^2^Chinese Pituitary Adenoma Cooperative Group, China Pituitary Disease Registry Center, Beijing, China; ^3^Department of Pathology, Chinese Academy of Medical Sciences and Peking Union Medical College, Peking Union Medical College Hospital, Beijing, China

**Keywords:** sellar and suprasellar region, central diabetes insipidus, germ cell tumor, langerhans cell histiocytosis, craniopharyngioma

## Abstract

**Purpose:** To increase knowledge for the early differential diagnosis and accurate therapeutic strategies for pediatric patients with sellar or suprasellar region (SSR) lesions who initially present with central diabetes insipidus (CDI).

**Methods:** This is a retrospective review of 55 pediatric patients (≤14 years old) with identified lesions in the SSR who initially presented with CDI at a large pituitary center between 2012 and 2018. The following data were summarized: demographic, clinical, endocrine, and neuroimaging data, intraoperative findings, histopathological findings, treatments, and prognosis.

**Results:** In our group, the etiologies of the SSR lesions included germ cell tumors (GCTs, 74.5%), Langerhans cell histiocytosis (LCH, 18.2%), and craniopharyngioma (CP, 7.3%). Almost all patients (50/55, 90.9%) showed anterior pituitary dysfunction [multiple axes dysfunction (38), and isolated axis dysfunction (14)], while the GH/IGF-I axis was the most affected. Most GCT patients presented with various clinical manifestations besides CDI and had elevated β-HCG, whereas LCH and CP patients mostly presented few non-specific symptoms besides CDI and most had normal level tumor markers. Sellar MRI demonstrated that posterior pituitary bright spot disappearance occurred in all patients, and pituitary stalk thickening was observed in 96.7% of patients. Treatment varied due to the different etiologies of the SSR lesions. After follow-up for 35.4 ± 20.2 months, the proportions of patients who needed AVP (arginine vasopressin) for GCT, LCH, and CP were 86.5, 100, and 75%, respectively, and the proportions of patients who needed HRT were 89.2, 50, and 75%, respectively.

**Conclusion:** For pediatric SSR lesions that first manifest as CDI, we should comprehensively consider clinical characteristics and imaging features to aid in their early differential diagnosis. Tumor markers and surgical histopathology are also great complements for the differential diagnosis. Additionally, various treatment strategies should be adopted according to different causes to improve the child's prognosis and quality of life.

## Introduction

Central diabetes insipidus (CDI), which is characterized by polyuria and polydipsia, is one of the most common and earliest symptoms of sellar and suprasellar lesions in children, and the reported incidence of CDI in these patients is ~42–80% (1). The intrinsic mechanism of CDI is believed to be related to the anatomical proximity of the lesions to the hypothalamic-pituitary axis, which disrupts the release or transportation of the arginine vasopressin (AVP), increasing urine output.

Pituitary adenoma is the most common cause of SSR masses in adults, followed by craniopharyngioma (CP), and meningioma (2, 3). However, in children, the type and frequency of SSR masses vary greatly, CP is the major cause of SSR masses, and pituitary adenoma is extremely rare, etiologies also includes tumors such as germ cell tumors (GCTs), gliomas; histiocytic diseases such as Langerhans cell histiocytosis (LCH); inflammatory diseases such as pituitary abscesses; and congenital diseases such as pituitary cysts (4). Among these etiologies, most can manifest as CDI, and GCT has been reported to be associated mostly with diabetes insipidus (DI) in 80% of children (5). However, few researches had systematically summarized pediatric SSR masses that initially manifest as CDI from the perspective of differential diagnosis. Therefore, we summarized all the pediatric cases of SSR lesions that initially manifested as CDI in Peking Union Medical College Hospital (PUMCH) from 2012 to 2018, aiming to improve the early differential diagnosis and facilitate timely and precise treatment options.

## Materials and Methods

### Patients

We retrospectively reviewed the PUMCH database to select all the inpatient children who had documented cases of lesions in the SSR with polydipsia and polyuria as initial symptoms between January 2012 and December 2018. We screened 5066 SRR masses patients and only 59 patients satisfied the inclusion criteria and 4 patients were excluded due to the exclusion criteria, and finally 55 patients were included in our study. All procedures involving human participants were performed in accordance with the ethical standards of the Institutional Ethics Committee of Peking Union Medical College Hospital at the Chinese Academy of Medical Sciences & Peking Union Medical College and with the 1964 Declaration of Helsinki and its later amendments or comparable ethical standards. Informed consent was obtained from all participants included in the study.

The inclusion criteria were as follows:

1. age ≤14 years old (4,899 not qualified);
2. polyuria and polydipsia as onset symptoms with a definitive diagnosis of CDI (78 not qualified, 58 without CDI and 20 with post-surgery diabetes insipidus);
3. MRI-confirmed sellar or suprasellar lesions (4 not qualified); and
4. operation and pathological confirmation (26 not qualified).

The exclusion criteria were as follows:

1. psychogenic polydipsia (*n* = 0) and
2. polyuria caused by kidney abnormalities or other non-sellar/suprasellar lesions (*n* = 4).

### Clinical Manifestation

1. CDI was defined as follows:
  - Twenty-four-hour urine output > 2 L/m^2^ (or ~150 ml/kg/d at birth, 100–110 ml/kg/d below 2 years old and 40–50 ml/kg/d after 2 years old), urine specific gravity (SG) < 1.005, urine osmotic pressure ≤ 200 mOsm/kg·H_2_O, and plasma osmolality ≥ 300 mOsm/kg·H_2_O (6);
  - Water deprivation test: dehydration symptoms after 4–12 h of water deprivation without a urine volume decrease, urine specific gravity <1.015, and urine osmotic pressure < plasma osmotic pressure (7);
  - Vasopressin test: rapid increase in urine specific gravity ≥1.018, urinary osmotic pressure rise > 9%, and urine osmotic pressure/plasma osmotic pressure >1 (7).
2. Short stature was defined as eight of 2 standard deviations (SDs) or more below the mean height for individuals of the same sex and chronological age in a given population or below the 3rd percentile (8).

### Perioperative Assessment

All patients underwent endocrinological assessments, which mainly included the measurement of serum growth hormone (GH), insulin-like growth hormone-1 (IGF-1), adrenocorticotropic hormone (ACTH), plasma cortisol (F), thyroid-stimulating hormone (TSH), free triiodothyronine (FT3), triiodothyronine (T3), free thyroxine (FT4), thyroxine (T4), prolactin (PRL), follicle-stimulating hormone (FSH), luteinizing hormone (LH), progesterone (P), testosterone (T) and estradiol (E2). And the reference ranges were as follow: GH <2 ng/ml, IGF varied according to ages, ACTH 0–46 pg/ml, F 4–22.3 2g/dl, TSH 0.38–4.34.IU/ml, FT3 1.8–4.1 pg/ml, T3 0.66–1.92 ng/ml, FT4 0.81–1.89 ng/dl, T4 4.3–12.5 2g/dl, PRL 2.64–13.13 ng/ml. For boys, reference ranges of sex hormones were FSH 1.27–19.26 IU/l, LH 1.24–8.62 IU/l, P 0.10–0.84 ng/ml, T 1.75–7.81 ng/ml, E2 < 47 pg/ml. And for girls, FSH 0.25–8.64 mIU/ml, LH 0.09–31.66 mIU/ml, P 0–0.73 ng/ml, E2 < 40 pg/ml, T 0.10–0.75 ng/ml. Hypothyroidism was diagnosed when serum FT4 and FT3 levels were below the normal range and serum TSH level was inappropriately low or normal; hypogonadism was diagnosed according to IGF-1 lower than normal range or peak serum GH level < 10 ng/ml in provocative test (insulin tolerance tests and L-arginine tests); gonadotropin deficiency mainly diagnosed according to serum FSH, LH and E2 level; ACTH deficiency was diagnosed as low morning cortisol or response to cortrosyn stimulation testing (9). All patients underwent the assessment of blood electrolytes, which mainly included serum sodium (135–145 mmol/L), potassium (3.5–5.5 mmol/L), and chloride (96–105 mmol/L). Tumor markers, including AFP and β-HCG, were assessed in the blood of 51 patients and cerebrospinal fluid (CSF) of 49 patients. We defined the normal range as serum AFP ≤ 20 ng/ml, serum β-HCG ≤ 5 IU/L, CSF AFP ≤ 0.605 ng/ml, and CSF β-HCG ≤ 5 IU/L.

The MRI scans were performed on the 1.5/3.0-Tesla system (GE, Co., Fairfield, Connecticut, USA). All patients had sagittal, coronal and axial MRI evaluations, which included T1-weighted imaging (T1WI) and T2-weighted imaging (T2WI). T1-weighted sequences were performed after intravenous injection of gadopentetate dimeglumine (Gd-DTPA) at a dose of 0.1 mmol/kg. The tumor volume was calculated according to the following formula: volume = sagittal × coronal × axial diameters × π /6 (mL) (10). The Knosp classification was used to evaluate cavernous sinus invasion of the sellar lesions on preoperative MRI. We also assessed the posterior pituitary bright spot, the position and shape of the optic chiasma and pituitary stalk and the tumor location. Pituitary stalk diameter was measured on sagittal and coronal images at the following positions (9): (1) proximal part of the pituitary stalk (near its origin from the median eminence), (2) distal part (at its insertion on the pituitary gland), and (3) at the level of maximum thickness along its length. The pituitary stalk was considered thickened if the maximum measurements on sagittal and coronal images was 3 mm or greater, and thickened stalks were graded as minimally thickened (3.0–4.5 mm), moderately thickened (4.5–6.5 mm), or severely thickened (more than 6.5 mm) (11). Besides, bright spot referred to the hyper-intensity of posterior pituitary observed on T1WI. All hyper/hypo-intensity were compared with intensity of normal gray matter. All the MRI data were analyzed by an experienced radiologist and an experienced neurosurgeon simultaneously.

### Surgery and Histopathology

Forty-three (78.2%) patients underwent transsphenoidal surgery (TSS), 6 (10.9%) underwent craniotomy, and 6 (10.9%) underwent bone marrow biopsy to clarify the histopathological diagnosis. All operations were performed by experienced neurosurgeons.

All surgical specimens were examined by hematoxylin-eosin (HE) staining and SSR mass routine staining sets that included p53, Ki-67%, CD117, PLAP, HCG, OCT3/4, CD30, CD1a, S100, and langerin analyses.

### Follow-Up

The follow-up evaluations included postsurgical anterior pituitary hormone findings, posterior pituitary function assessment and sellar MRI findings. Individualized therapy was based on histopathological diagnosis and included chemotherapy, radiotherapy, AVP intake and HRT (including GH, thyroxine, and corticosteroid and sex hormone).

## Results

### Demographics

A total of 55 patients were included in our study, with an average age of 10.3 ± 2.7 years. Among them, GCT, LCH, and CP accounted for 74.5, 18.2, and 7.3% of lesions, respectively. Except for LCH, which had an equal ratio between females and males, both the GCT and CP groups comprised more females than males. The mean interval from initial onset to diagnosis was 22.9 ± 24.3 months, and CP showed the longest disease duration, indicating the difficulty of diagnosing CP (though with no statistical significance). The average follow-up time was 36.6 ± 19.3 months. Detailed baseline characteristics are shown in Table 1.

**Table 1:** Demographic data for the 55 patients with different causes of CDI.

**Cause of CDI**	**No. of patients (%)**	**Sex (M/F)**	**Age (yr.)**	**Disease Duration (mo.)**	**Follow-up period (mo.)**
GCT	41 (74.5)	11/30	9.9 ± 2.5	21.5 ± 21.2	35.4 ± 20.2
LCH	10 (18.2)	5/5	11.2 ± 3.3	23.7 ± 30.8	43.4 ± 16.0
CP	4 (7.3)	1/3	11.4 ± 2.3	35 ± 31.3	32.5 ± 12.6
Total	55 (100)	17/38	10.3 ± 2.7	22.9 ± 24.3	36.6 ± 19.3

*CDI, central diabetes insipidus; GCT, germ cell tumor; LCH, Langerhans cell histiocytosis; CP, craniopharyngioma; yr, years; mo, month*.

### Clinical Manifestations

Children with SSR masses that initially presented with CDI could have additional accompanying non-specific symptoms beyond polyuria and polydipsia; among these symptoms, short stature, and headache were the most common. Besides, the majority (50/55, 90.9%) of our cohort presented with APD (anterior pituitary dysfunction), growth factor deficiency was the most frequent. Detailed information is provided below (Table 2). In addition, hyperprolactinemia occurred in 63.4, 30, and 25% of GCTs, LCHs, and CPs, respectively, and the incidence of electrolyte disturbance was 24.4, 30, and 0%, respectively.

- GCT: Patients were more likely to have additional manifestations (38/41, 92.7%); short stature (20/41, 48.7%) was most common, followed by headache (20/41, 48.7%) and inappetence (19/41, 46.3%). Additionally, 10 patients developed visual defects. Several children had deficiencies in pubertal development, with 3 boys having precocious puberty (6.6, 7.8, and 7.8 years old) and 3 girls experiencing amenorrhea (14.7, 13, and 13.9 years old), beyond which none of the girls had started menstruating (9.48 ± 2.05). In addition, 95.1% (39/41) of the children had APD, including multiple axes dysfunction (31/39, 79.5%) and isolated axis dysfunction (7/39, 17.9%). And for electrolyte disturbance, 2 with hypernatremia, 7 with hyponatremia.
  Except for 2 patients who could not undergo lumbar punctures, all children underwent examinations of serum and CSF tumor markers, including AFP and β-HCG. A total of 22.0% (9/41) of the children showed elevated serum β-HCG levels (higher than 100 IU/L in two cases and an average of 31.0 ± 22.2 IU/L for the other 7 cases). A total of 64.1% (25/39) of the children had CSF β-HCG levels that exceeded the threshold (higher than 100 IU/L in 3 cases and an average of 13.3 ± 7.6 IU/L for the other 22 cases). Only 5 patients had elevated β-HCG in both the serum and CSF. Two patients (4.9%) were found to have AFP levels higher than the threshold in both the serum and CSF, while the AFP levels of the others were all in the normal range. Subsequent histopathology analysis confirmed that one lesion was a germinoma and the other was a mixed GCT.
- LCH: There were 10 patients in this group, and they had fewer additional clinical manifestations; headache (5/10), short stature (3/10) and nausea (3/10) were the three most common symptoms. In addition, 1 patient had visual field defects, and 1 girl (14 years old) had amenorrhea. APD occurred in 80% of LCH cases, including multiple axes dysfunction (3/10) and isolated axis dysfunction (5/10). Besides, 2 patients manifested as hypernatremia and 1 as hyponatremia.
  Additionally, 9 patients presented with extrasellar lesions, and bone lesions were the most common (7/9), followed by thyroid (5/9), lung (4/9), and skin (1/9) lesions. Among them, 5 patients had multiple extrasellar system involvement, three had only SSR and bone involvement, and one only had lesions in the SSR and thyroid. None of these extrasellar lesions were associated with any specific manifestations.
  Nine patients underwent examinations for tumor markers, and 3 patients had slight elevations in their CSF β-HCG levels: 7 IU/L, 6 IU/L, and 6 IU/L (the threshold for CSF β-HCG was 5 IU/L). And the level of CSF AFP, serum AFP and serum HCG of these 3 patients were all in normal range. The other 6 patients all had normal level AFP and b-HCG.
- CP: These patients also had fewer additional manifestations: only short stature (2/4) and a visual acuity defect (1/4). Three of four CP cases had accompanying APD, and all presented with multiple axes dysfunction (Table 2). No patient showed electrolyte disturbance.
  Only 1 patient underwent an examination for tumor markers, all of which were within the normal range. The other 3 patients did not undergo examinations for tumor markers, as they were considered most likely to be CP.

**Table 2:** Anterior pituitary function in 55 patients.

**Anterior pituitary function**	**Number of patients**					
	**GCT**		**LCH**		**CP**	
Multiple axes dysfunction		31		3		3
GH + TSH + ACTH + Gonadotropin	6		1		0	
GH + TSH + ACTH	12		0		1	
GH + TSH + Gonadotropin	0		1		0	
GH + TSH	5		0		1	
GH + ACTH	3		0		0	
GH + Gonadotropin	3		1		0	
TSH + ACTH	1		0		0	
TSH + Gonadotropin	1		0		0	
ACTH + Gonadotropin	0		0		1	
Isolated axis dysfunction		8		5		0
GH	7		3		0	
TSH	1		0		0	
Gonadotropin	0		2		0	
Normal function		2		2		1

*GH, growth hormone; TSH, thyroid-stimulating hormone; ACTH, adrenocorticotropic hormone*.

### MRI Characteristics

Our cohort showed commonalities in MRI. The posterior pituitary bright spot disappeared in all patients, and 94.5% presented with a thickened pituitary stalk (78.0% in GCT, 100% in LCH). Additionally, 96.4% (53/55) of the SSR masses involved the suprasellar region (Table 3).

**Table 3:** MRI characteristics of different types of SSR lesions.

		**GCT**	**LCH**	**CP**
**SIZE**				
	**Max diameter**			
	≤1 cm	3 (7.3%)	4 (40%)	0
	1–2 cm	27 (65.9%)	6 (60%)	3 (75%)
	2–3 cm	6 (14.6%)	0	0
	≥3 cm	5 (12.2%)	0	1 (25%)
**MAX KNOSP GRADE**				
	0	29 (70.7%)	8 (80%)	3 (75%)
	I	3 (7.3%)	0	0
	II	6 (14.6%)	1 (10%)	1 (25%)
	III	1 (2.4%)	1 (10%)	0
	IV	2 (4.9%)	0	0
**INTENSITY**				
	**T1**			
	Iso	36 (87.8%)	7 (70%)	0
	Hyper	0	3 (30%)	3 (75%)
	Hypo	5 (12.2%)	0	1 (25%)^*1^
	**T2**			
	Iso	30 (73.2%)	8 (80%)	0
	Hyper	9 (21.9%)	1 (10%)	4 (100%)
	Hypo	2 (4.9%)	1 (10%)	0
**ENHANCEMENT**				
	Heterogeneous	21 (51.2%)	0	4 (100%)
	Homogeneous	20 (48.8%)	10 (100%)	0
**POSTERIOR PITUITARY BRIGHT SPOT**				
	Disappeared	41 (100%)	10 (100%)	4 (100%)
	Present	0	0	0
**STALK**				
	Measurements^*2^**(thickening indicates more than 3 mm)**			
	<3.0 mm	2 (73.2%)	0	0
	3.0–4.5 mm	6 (14.6%)	6 (60%)	0
	4.5–6.5 mm	12 (29.3%)	4 (40%)	0
	>6.5 mm	14 (34.1%)	0	1 (25%)
	Not observed	7 (17.1%)^*3^	0	3 (75%)^*4^
	Deviation from midline	13 (31.7%)	6 (60%)	–^*4^
**OPTIC CHIASM**				
	Uplift	20 (48.8%)	0	3 (75%)
	Normal	21 (51.2%)	10 (100%)	1 (25%)
**LOCATION**				
	Suprasellar	13 (31.7%)	3 (30%)	2 (50%)
	Sellar	2 (4.9%)	0	0
	Suprasellar and sellar	26 (63.4%)	7 (70%)	2 (50%)

*1. *This case showed T1 central hypointensity and a rim hyperintensity*.

*2. *Measurements indicated the average value of the maximum measurements on sagittal and coronal images*.

*3. *Among GCT patients, 7 (17.1%) pituitary stalks were not observed as being surrounded by large tumors*.

*4. *Among CP patients, 3 (75%) pituitary stalks were not observed as being surrounded by tumor. One (25%) presented with a thickened pituitary stalk that deviated from the midline*.

**Figure 1:**
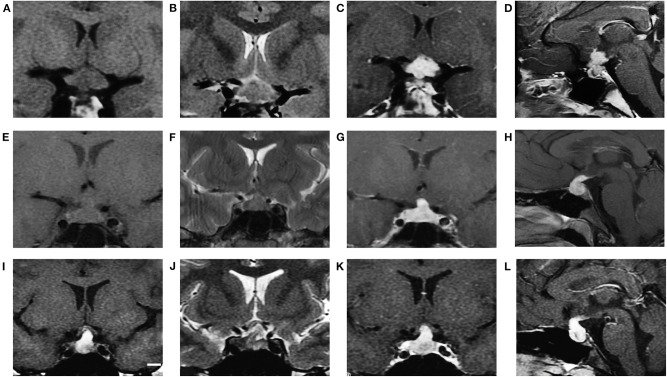
MRI characteristics of GCT, LCH and CP (cases 7, 46, and 53, respectively). GCT showed isointensity on T1WI (**A** coronal), iso to hyperintensity on T2WI (**B** coronal) and obvious enhancement after gadolinium injection (**C** coronal, **D** sagittal). LCH showed isointensity on T1WI (**E** coronal), isointensity on T2WI (**F** coronal) and obvious enhancement after gadolinium injection (**G** coronal, **H** sagittal). CP showed hyperintensity on T1WI (**I** coronal), hyperintensity on T2WI (**J** coronal) and obvious heterogeneous enhancement (**K** coronal, **L** sagittal).

We found significant differences in the different types of SSR lesions (Figure 1).

- All GCTs presented T1WI hypo/isointensity (hypo: iso = 5:36), and 95.1% presented T2WI iso/hyperintensity (iso: hyper = 30:9). Except for one case, which received radiotherapy and chemotherapy before surgery and showed slight enhancement, all other cases showed obvious enhancements, and the ratio of heterogeneous enhancement to homogeneous enhancement was 21/20. In addition, 20% (9/41) of cases had accompanying with pineal body lesions.
- Most cases of LCH (60%) had isointensity on T1WI and T2WI. The other 4 showed iso/hyperintensity on T1WI with no certain pattern on T2WI. Ten cases showed obvious and homogeneous enhancement.
- The majority (3/4) of CP cases showed hyperintensity on T1WI and T2WI, as well as heterogeneous enhancement. The other case had diameters > 3 cm and presented T1 central hypointensity and rim hyperintensity and T2 hyperintensity and rim enhancement with central low attenuation.

### Intraoperative Findings and Histopathologic Results

**Figure 2:**
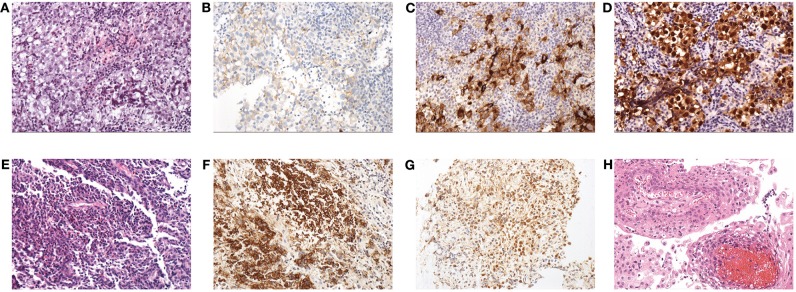
Histopathological findings: **(A)** HE staining (×200) of GCT; note the diffuse tumor cells that aggregated into sheets, with a large, partially transparent cytoplasm and obvious nucleoli. Stroma was scant and mainly comprised of lymphocytes. **(B)** Expression of CD117 in GCT. **(C)** Expression of PLAP in GCT. **(D)** Expression of OCT3/4 in GCT. **(E)** HE staining (×200) of LCH; note that large amounts of mononuclear phagocytes are diffusely distributed and intermingled with eosinophilic granulocytes and lymphocytes. **(F)** Expression of CD1a in LCH. **(G)** Expression of S100 in LCH. **(H)** HE staining (×200) of CP; the squamous epithelium with papillary hyperplasia and the fibrous vessel axis can be seen.

- Regarding GCT, the gross appearance was usually gray-white with a fish-meat pattern. Regarding the histological classification, 40 patients had germinomas, and only one had non-germinomatous GCT (NGGCT), which was a mix of germinoma and teratoma. The immunohistochemical results showed positivity for CD117, PLAP or OCT3/4 in all cases (Supplementary Table 1, Figure 2). A total of 75.6% (31/41) of the patients had data for the ki-67%, and the average value was 47.9% ± 21.7%.
- Regarding LCH, 70% (7/10) of the patients underwent bone biopsies to inform the diagnosis. Only 30% (3/10) underwent TSS to obtain the tissue from the sellar or suprasellar lesions, and the gross appearance was gray-white and rubbery. The histopathological results showed positivity for CD1a and S100 in all cases (Figure 2). A total of 50% of biopsy tissue had data for the ki-67%, and the average value was 5.6% ± 5.4%.
- Regarding CP, the gross appearance tended to be oil-like and solid tissue mixed with cystic fluid, but 1 case had a gray-white and soft-tissue appearance. Histopathology mainly indicated cholesterol crystals and inflammatory cells (Figure 2).

### Treatment Outcomes and Follow-Up

**Figure 3:**
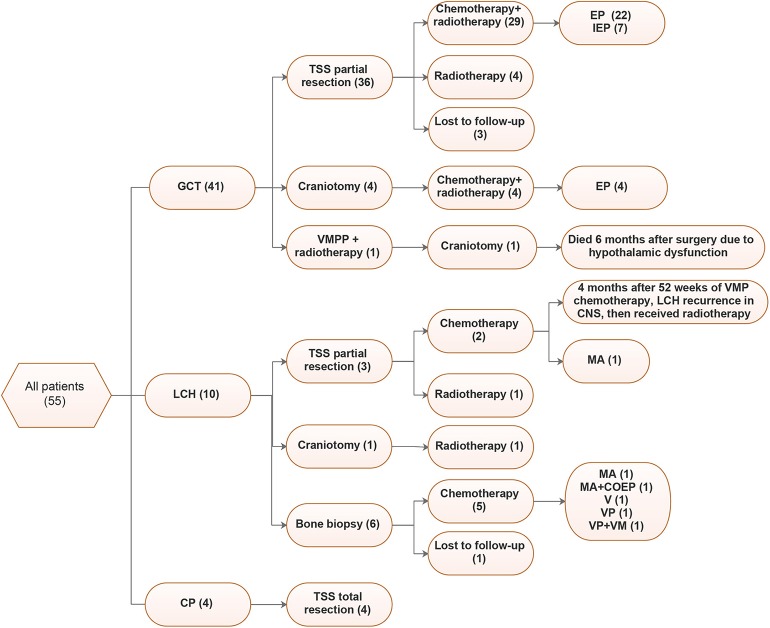
Treatments of 55 patients. VMPP, vincristine (V) + methotrexate (M) + pingyangmycin (P) + Cisplatin (P); EP, Etoposide + Cisplatin; IEP, Isophosphoramide (I) + Etoposide (E) + Cisplatin (P), MA, methotrexate (M) + Ara-c (A); VMP, Vindesine (V) + Methotrexate (M) + Prednisone (P); VM, Vindesine (V) + Methotrexate (M); VP, Vinblastine (V) + Prednisone (P).

Figure 3 shows the detailed treatments of the patients in our cohort. Postsurgical therapy varied for different lesion types.

For GCT, 80.5% (33/41) of cases underwent tumor partial resections through TSS, among which 29 received postsurgical chemotherapy and radiotherapy and 4 only received radiotherapy. Additionally, 4 patients underwent craniotomy, followed by radiotherapy. EP (Etoposide+Cisplatin) was the most common chemotherapy regimen, accounting for 26/33 cases (78.8%), followed by IEP (Isophosphoramide + Etoposide + Cisplatin, 7/33, 21.2%). Although most LCH cases underwent bone biopsies, only three underwent TSSs. Chemotherapy was the main treatment for LCH, and in our cohort, 77.8% (7/9) received chemotherapy, the regimens varied, and MA (methotrexate + Ara-c) was more common. For CP, all patients underwent total tumor resection.

For GCT, 3 patients were lost to follow-up, and 1 patient died 6 months after surgery due to hypothalamic dysfunction. The other 37 patients had good control of the disease without relapse. All patients showed good recovery from headache, inappetence, nausea, and other clinical symptoms. However, recovery in terms of CDI, height, pubertal development and visual defects was worse, with recovery rates of 10.8% (4/37), 64.9% (24/37), 24.3% (9/37), and 10% (1/10), respectively. At the last follow-up, 1 recovered from CDI and didn't need HRT, 3 recovered from CDI but still needed HRT, 4 only needed AVP supplementation, and 29 needed long-term AVP supplementation and HRT. The most common HRT was corticosteroid (90.6%, 29/32), followed by thyroxine (78.1%, 25/32), GH (28.1%, 9/32), and sex hormone (15.6%, 5/32).

For LCH, 1 patient was lost to follow-up, and 1 patient relapsed 6 months after VMP chemotherapy and then received hypothalamic-pituitary-3rd ventricle radiotherapy. The other 8 patients all had tumor regression after the treatment.

The recovery rates for CDI, height, pubertal development and visual defects were 0% (0/9), 66.7% (6/9), 33.3% (3/9), and 0% (0/1), respectively. At the last follow-up, 2 patients needed long-term HRT and AVP supplementation, and 7 patients only needed AVP supplementation. The most common HRT was thyroxine (100%, 2/2), followed by corticosteroid (50%, 1/2).

For CP, 4 patients all had good control of the disease, and none relapsed.

The recovery rates of CDI, height, pubertal development and visual defects were 25% (1/4), 50% (2/4), 25% (1/4), and 0% (0/1), respectively. At the last follow-up, 1 recovered from CDI but still needed HRT, 1 needed AVP only, and 2 needed AVP and HRT. Corticosteroid and thyroxine were the most common HRT regimens (both 66.7%, 2/3).

## Discussion

CDI, which is characterized by polyuria and polydipsia, is usually the first symptom of SSR lesions but is easily ignored. Approximately three-quarters of our patients had symptoms or signs for more than 6 months before their diagnoses, and the diagnostic delay could be up to 10 years. Thus, more attention should be paid to the early detection and diagnosis of SSR lesions with CDI. However, we still lack useful data for differential diagnoses, thus impeding timely and precise therapy.

Previous studies about pediatric CDI mainly focused on etiology and common features, while our study mainly focused on CDI caused by SSR lesions, and summarized the detailed clinical manifestations, tumor markers, radiology characteristics and histopathology about each etiology, trying to offer evidence for diagnosis and differential diagnosis. Acquired infiltrative disease or tumor including CP, GCT, LCH, pituitary adenoma, lymphoma, astrocytoma, and cavernous hemangioma were all the etiology of CDI reported before (9, 11–14). In our cohort, the causes of SSR lesions only included GCT, LCH and CP, whose proportions were 74.5, 18.2, and 7.3%, respectively. Liu et al. (14) also found that GCT was the most common cause (52.6%) among acquired infiltrative disease or tumor in Taiwanese children with CDI, which was consistent with our results. This might indicate that GCT would be the most likely to invade the hypothalamic-pituitary axis. While in studies of Werny et al. (9), Maghnie et al. (11), Catli et al. (13), the proportion of GCT among acquire infiltrative disease or tumor was much lower (21.3, 21.1, and 26.7%, respectively). Considering that PUMCH is both a general hospital and the center for managing rare and complicated disorders, patients with endocrine problems more commonly visit this hospital, so the higher prevalence of GCT and less variety of the etiology in our study might be explained by potential selection bias.

Presurgical diagnoses of SSR masses should comprehensively consider clinical manifestations, tumor markers and MRI characteristics.

- We suggest that for children with SSR masses with CDI at onset, we should consider GCT when the following situations occur: (1) CDI accompanied with short stature, headache, delayed sexual development, precocious puberty and other symptoms; previous studies also supported our results (15) (2) elevation in β-HCG in the blood or CSF (>5 IU/l), besides, a high level of AFP possibly indicating a non-germinomatous GCT component; (3) MRI showing T1 hypo/isointensity, T2 iso/hyperintensity, and typically homogeneous enhancement or heterogeneous enhancement with cysts or hemorrhage, which is consistent with previous studies (16, 17). Additionally, enhancement in the pineal body, which was thought to be another common site for GCT in children, would assist in the diagnosis (18).
  The sensitivity of serum β-HCG and CSF β-HCG in our study was 22.0 and 64.1%, respectively, and Qaddoumi et al. (19) found that the level of β-HCG was higher in CSF than serum (58.8% > 41.2%). However, their sensitivity of serum β-HCG was obviously higher than ours, which might be due to their higher proportion of GCT metastases.
- LCH was less common than GCT in our cohort, and the following conditions can help its diagnosis. (1) There are few non-specific symptoms beyond CDI, with headache being the most common; (2) MRI typically shows isointensity in both T1WI and T2WI (though with low specificity) and obvious homogeneous enhancement. Although previous studies have indicated that MRI of LCH in the hypothalamic-pituitary region showed no specific pattern, homogeneous enhancement was usually obvious (20, 21); (3) Extrasellar lesions, especially nodules in the bone, would be suggestive; (4) Tumor markers such as AFP and β-HCG are in the normal range or slightly elevated.
- Although CP is the most common cause of SSR masses in children, it does not usually present as CDI, with one report showing that ~15% of pediatric CP patients present with CDI (22). Therefore, CP was less common in our cohort, and we considered CP when the following situations occurred: (1) fewer nonspecific symptoms, with short stature being the most common; previous study indicated that common symptoms of CP were usually non-endocrine related, such as headache and visual disturbances (11). Given the small amount of CP in our group, differences could be explained. (2) MRI typically presents with hyperintensity on T1WI and T2WI and heterogeneous enhancement, but the solid component could be variable on T2WI; (3) tumor markers such as AFP and β-HCG were in the normal range, with possible exclusion of CP when tumor marker levels are high.

However, it was sometimes impossible to confirm the diagnosis based only on clinical manifestations, tumor markers and MRI characteristics. Obtaining pathological evidence through surgical biopsy was of great significance for accurate diagnoses and for subsequent decisions regarding which therapy to choose. For different types of SSR lesions, treatment and long-term outcomes varied greatly.

- The guidelines suggest that patients with clinically suspicious intracranial GCT should undergo examination for CSF and serum tumor markers after contraindications are ruled out. Biopsy should be performed for pathological confirmation if AFP or β-HCG (serum or CSF) levels are below the threshold. Patients with consistent radiological imaging and AFP and/or β-HCG (serum or CSF) above the thresholds could begin therapy instead of having a surgical biopsy (23). However, in our group, all patients underwent biopsies, and when GCT was confirmed by intraoperative rapid pathology, patients received partial resections and postsurgical chemotherapy or radiotherapy. We sometimes found it difficult to distinguish intracranial GCT from LCH on MRI. Additionally, slight elevations in tumor markers could not rule out LCH, as the CSF β-HCG levels of 3 patients were slightly above the threshold in our study. Furthermore, regarding the long-term prognoses with different therapies for SSR lesions in children, a precise diagnosis is of great significance. Therefore, for children who are suspected of having GCT, a biopsy is necessary if tissue for pathology is accessible through a minimally invasive method like TSS.
  In 33 GCT patients who underwent partial tumor resections by TSS, 87.9% received chemotherapy and radiotherapy and 12.1% received radiotherapy only. We found no significant differences in the survival rate, CDI recovery rate, HRT rate, height increase rate or pubertal development rate between the two therapies (96.6 vs. 100%, *P* = 1; 86.2 vs. 100%, *P* = 1; 86.2 vs. 75%, *P* = 0.50; 65.5 vs. 50%, *P* = 0.61; and 24.1 vs. 25%, *P* = 1, respectively). According to experiences in our center and previous studies, radiotherapy was the main therapy for intracranial GCT, while combination therapy with chemotherapy would not improve the long-term prognosis. However, chemotherapy could effectively reduce the dose of radiotherapy, thus reducing the risk of neuropsychological sequelae caused by irradiation of the developing brain (23, 24). In addition, combination therapy showed a better overall survival rate and disease-free survival rate in acute and severe patients and a lower HRT rate in pediatric patients (24, 25). Therefore, in our study, combined chemoradiotherapy was the major treatment. To determine whether chemotherapy combined with radiotherapy can reduce the effects on the children's pubertal development, further follow-up and large-scale studies are needed.
- For patients with suspected LCH, biopsy is necessary, as LCH may be confused with GCT or other SSR lesions. The diagnosis should also be based on histological and immune-phenotypic examinations beyond clinical manifestations and MRI features, which is supported by previous reports (20, 26). The main therapy for intracranial LCH is chemotherapy, while a particular regimen should be based on the location and presentation of the lesions. In addition, SSR lesions and any brain lesions, except for isolated skull vault lesions, are the indication for systemic therapy (26). A standard regimen with VP (Vinblastine + Prednisone) is known to be effective in this situation (20, 21, 26). Improvements in the neurological condition with the use of Ara-c, methotrexate and intravenous immunoglobulin have been reported (27–30). Some previous reports have also suggested that treatment with 2-CdA, etoposide, or radiation soon after DI onset may reverse the condition (31–35). Consistently, in our group, VP and MA (methotrexate + Ara-c) were both associated with beneficial outcomes.
  At the last follow-up, all patients suffered from CDI, though the needs for AVP varied, which is consistent with the opinions of experts (26). Fifty percent still needed HRT, and hypothyroidism was the most common symptom. However, Fahrner et al. (36) found that GH deficiency was the most frequent symptom in children, followed by hypothyroidism. We thought the differences might because most of our LCH patients had already completed GH therapy at the last follow-up, as the average age of our patients was higher than that of Fahrner's study (11.2 vs. 5 years).
- For CP, all 4 patients underwent total tumor resections through TSS. However, the best treatment for children with CP is still controversial. Some believed that with the improvement of surgical techniques, complete resection can be achieved without increasing complications or deaths (37, 38), thus making it the optimal choice. Others insisted that partial resection followed by radiotherapy would be safer for children and that the rate of obesity could be reduced, which was confirmed by some small retrospective studies (39, 40). However, it seemed to be inevitable that CP patients will develop deficiencies in pituitary function after surgery, and previous studies (39, 41–43) showed that pituitary insufficiency could be present in almost 100% of long-term survivors. Thus, further efforts should be invested to monitor and maintain normal hormone levels, thereby improving quality of life.

Our study has limitations. First, given it was a retrospective observational study, data collection may be limited and prevent us from coordinating a standardized assessment of each patient. Second, limited time of follow-up could cause difficulty and bias in studying the short and long term outcomes, such as radiotherapy induced hypopituitarism may have been missed in a vast majority of patients. Third, we defined GH deficiency mainly based on GH and IGF-1, but the accurate definition of GH deficiency required dynamic tests. Only 12 patients in our study performed dynamic tests at the time of diagnosis and 23 people underwent dynamic tests during the follow-up, so we possibly underestimated GH deficiency, which could cause inaccuracy in our results. Fourth, our patients were enrolled from a national referral center, which might introduce potential selection bias. A large-scale prospective multi-center case-control study is warranted to address these limitations.

## Conclusion

For pediatric SSR masses that initially present as CDI, the assessment of clinical manifestations and the measurement of anterior pituitary function and tumor markers in serum or CSF are important for the diagnosis and differential diagnosis of these lesions. MRI is also an effective tool for the diagnosis of SSR lesions. For SSR masses in children with normal or slightly elevated levels of tumor markers, biopsy is an effective method to achieve a definitive diagnosis and provides objective pathological evidence to determine precise treatment strategies and further improve the patient outcomes and quality of life.

## Data Availability Statement

All datasets generated for this study are included in the article/Supplementary Material.

## Ethics Statement

The studies involving human participants were reviewed and approved by The Institutional Ethics Committee of Peking Union Medical College Hospital at the Chinese Academy of Medical Sciences & Peking Union Medical College. Written informed consent to participate in this study was provided by the participants' legal guardian/next of kin. Written informed consent was obtained from the individual(s), and minor(s)' legal guardian/next of kin, for the publication of any potentially identifiable images or data included in this article.

## Author Contributions

XJ, ZW, and BX contributed conception and design of the study. WW, LG, XG, and CF performed the data curation and analysis. KD and WL analyzed and interpreted the results. XJ, ZW, and BX drafted and reviewed the manuscript. All authors read and approved the final manuscript.

### Conflict of Interest

The authors declare that the research was conducted in the absence of any commercial or financial relationships that could be construed as a potential conflict of interest.
