# Supplementary material for: Clinical Characteristics of Pediatric Patients With Sellar and Suprasellar Lesions Who Initially Present With Central Diabetes Insipidus: A Retrospective Study of 55 Cases From a Large Pituitary Center in China
Source: Front Endocrinol (Lausanne). 2020 Feb 20;11:76. doi: 10.3389/fendo.2020.00076 (PMC7044264; doi:10.3389/fendo.2020.00076)
Supplement: Supplementary file 1 [file Table_1.DOCX]

| Supplementary Table 1. Clinical characteristics of the 55 CDI patients with sellar or suprasellar masses | | | | | | | | | | | | | | | |  |
| --- | --- | --- | --- | --- | --- | --- | --- | --- | --- | --- | --- | --- | --- | --- | --- | --- |
| No | Age/sex | Tumor type | Course (mo.) | Other Clinical manifestation | Growth  (BA: yr  Ht: cm) | Ophthalmic examination | Endocrine examination | Tumor Marker* | Opera-tion | Intraoperat-ive findings | | Histo-pathology | Follow-up (mo.) | | Outcomes |  |
| 1 | | 9.8/F | GCT | 24 | Headache, nausea and vomiting, inappetence, growth retardation | BA:8-9  Ht:122 (<3%) | - | GH, gonadotropic deficiency，PRL↑ | CSF: β-HCG+ | TSS | Grey-white, rubbery, fish-meat like | | CD117+, PLAP+,  OCT3/4+, β-HCG+,  Ki-67：50% | 45.3 | | Tumor partial resection, 4 EP chemotherapy, radiotherapy, HRT(D+C+G+T+E)height increase，delayed puberty |
| 2 | | 10.3/M | GCT | 12 | Growth retardation | BA:5-6  Ht:133(3%-10%) | - | GH, gonadotropic deficiency, PRL↑ | CSF: β-HCG+ | TSS | Grey-white, rubbery, fish-meat like | | HCG-, CD117+, PLAP+, OCT3/4+  ki-67: 50% | 16.3 | | Tumor partial resection, 4 EP chemotherapy, radiotherapy, HRT(D+C+T), no height increase but normal pubertal development |
| 3 | | 10.2/F | GCT | 48 | Growth retardation | BA:7-8  Ht:128(<3%) | - | GH, corticotropic deficiency | CSF: β-HCG+ | TSS | Grey-white, rubbery, fish-meat like | | P53+, CD117+, PLAP+,  ki-67: 50% | 63.7 | | Tumor partial resection, 4 EP chemotherapy, radiotherapy, HRT(D+C+G+T+E)， height increase, delayed puberty |
| 4 | | 14.7/F | GCT | 20 | Amenorrhoea | Ht:149.5(3-10%) | - | GH deficiency, PRL↑ | - | TSS | Grey-white, rubbery, fish-meat like | | CD117±, PLAP+, OCT3/4+,  Ki-67: 10% | 34.5 | | Tumor partial resection, 4 IEP chemotherapy, radiotherapy, HRT(D)，no height increase, pubertal developed |
| 5 | | 10.7/F | GCT | 120 | Headache, nausea, growth retardation | BA:8-9  Ht:127(<3%) | - | Panhypo, PRL↑, electrolyte disturbance | Blood: CEA+ | TSS | Grey-white, rubbery, fish-meat like | | CD117+, PLAP+, OCT3/4+ | 49.9 | | Tumor partial resection, 4 EP chemotherapy, radiotherapy, HRT(D+C+G+T)，height increase , delayed puberty |
| 6 | | 13.3/M | GCT | 4 | Inappetence | BA:14-16  Ht:158(50-75%) | - | GH, gonadotropic deficiency, PRL↑ | CSF: β-HCG+ | TSS | Grey-white, rubbery, fish-meat like | | HCG-, CD117+, PLAP+, OCT3/4+, CD30+,  Ki-67: 50% | 49.0 | | Tumor partial resection, 6 EP chemotherapy, radiotherapy, HRT(D+C+T)，height increase , delayed puberty |
| 7 | | 12.7/ F | GCT | 10 | Headache, inappetence, | NA | Binocular VA Panhypo impairment, upper VF defects | GH, thyrotropic,  corticotropic deficiency | - | TSS | Grey-white, fish-meat like | | P53-, β-HCG-, CD117±, PLAP+  Ki-67: 10% | 26.2 | | Tumor partial resection, radiotherapy, HRT(D+C+T)，no height increase, delayed puberty |
| 8 | | 12.8/F | GCT | 24 | Headache, nausea and  vomiting, growth retardation | BA:8-11  Ht:129.8(<3%) | - | GH deficiency | - | TSS | Grey-white, little soft, fish-meat like | | CD117+, PLAP+  Ki-67: 30% | 67.2 | | Tumor partial resection, 4 EP chemotherapy, radiotherapy，height increase and normal pubertal development |
| 9 | | 10.4/F | GCT | 11 | Inappetence | BA:7-9  Ht:147.5(75-97%) | - | GH, thyrotropic,  corticotropic deficiency | - | TSS | Grey-white, soft, fish-meat like | | P53+, β-HCG-, CD117+, PLAP+, OCT3/4+,  Ki-67: 60% | 6.3 | | Tumor partial resection, 4 EP chemotherapy, radiotherapy, HRT(D)，no height increase or pubertal development |
| 10 | | 11.8/F | GCT | 3 | Inappetence, growth retardation | BA:10  Ht:138(3-10%) | Binocular VA panhypo impairment | GH, thyrotropic,  corticotropic deficiency | CSF: HCG+ | TSS | Dark red, rubbery | | β-HCG±, CD117+, PLAP+  Ki-67: 30% | NA | | NA |
| 11 | | 13.0/F | GCT | 13 | Nausea and vomiting, amenorrhoea | BA:16-17  Ht:170(>97%) | - | Panhypo, PRL↑ | Blood: AFP+&HCG+ | TSS | Grey-white, fish-meat like | | HCG-, CD117+, PLAP+  Ki-67: 30% | 55.4 | | Tumor partial resection, 6 EP chemotherapy, radiotherapy, HRT(D+C+T+E)，normal height and  pubertal development |
| 12 | | 6.3/F | GCT | 6 | Nausea and vomiting, hypodynamia, inappetence, growth retardation | BA:3-5  Ht:105(<3%) | - | Panhypo, PRL↑ | CSF: HCG+ | TSS | Grey-white, rubbery, fish-meat like | | CD117+, PLAP+, OCT3/4+,  Ki-67: 25% | 26.7 | | Tumor partial resection, 3 IEP chemotherapy, radiotherapy, HRT(D+C+T)，height increase and pubertal development |
| 13 | | 13.9/F | GCT | 60 | Hypodynamia, amenorrhea | NA | Binocular blindness, mydriasis, limited right-eye movements in adduction | GH, thyrotropic,  corticotropic deficiency | - | Crani-otomy | Grey-white mixed with brown, rubbery | | HCG-, CD117+, PLAP±, OCT3/4+,  Ki-67: 90% | 23.1 | | Tumor subtotal resection, 2 EP chemotherapy, radiotherapy, HRT(D+C+T)，no height increase or pubertal development |
| 14 | | 13.0/M | GCT | 24 | Hypodynamia | Ht:161(25-50%) | - | Panhypo, PRL↑, electrolyte disturbance | CSF: HCG+ | TSS | Grey-white, rubbery, fish-meat like | | CD117+, PLAP+ | 33.9 | | Tumor partial resection, radiotherapy, HRT(D)，height increase but delayed puberty |
| 15 | | 11.3/F | GCT | 14 | Headache, nausea and vomiting, growth retardation | BA:9-10  Ht:133(<3%) | Binocular VA panhypo impairment  bitemporal VF defects | GH, thyrotropic,  corticotropic deficiency, PRL↑ | CSF: HCG+ | TSS | Grey-pink, rubbery | | CD117+, PLAP+, OCT3/4+,  Ki-67: 40% | 33.2 | | Tumor subtotal resection, 4 EP chemotherapy, radiotherapy, HRT(D+C+T)，height increase but delayed puberty |
| 16 | | 6.6/M | GCT | 16 | Growth of pubes | NA | - | Thyrotropic,  corticotropic deficiency, PRL↑ | Blood: HCG+  CSF: HCG+ | TSS | Grey-white, rubbery, fish-meat like | | HCG+, CD117+, PLAP+, OCT3/4+,  Ki-67: 80% | 20.9 | | Tumor partial resection, 6 EP chemotherapy, radiotherapy, HRT(D)，normal height and pubertal development |
| 17 | | 14.9/M | GCT | 13 | Headache, hypodynamia, inappetence | BA:13-15  Ht:169(25-50%) | - | Panhypo, PRL↑ | CSF: HCG+ | TSS | Grey-red, soft | | P53-, HCG+, CD117+, PLAP+, OCT3/4+, S100+  Ki-67: 60% | 39.5 | | Tumor partial resection, 2 EP chemotherapy, radiotherapy, HRT(D+T+A)，height increase and  pubertal development |
| 18 | | 6.9/F | GCT | 14 | Hypodynamia, growth retardation | NA | - | GH deficiency | - | TSS | Grey-white, little soft | | P53-, PLAP+, CD117+  Ki-67: 1% | 34.8 | | Tumor partial resection, EP chemotherapy, radiotherapy, HRT(G)，height increase, delayed puberty |
| 19 | | 7.7/F | GCT | 7 | Headache, inappetence, | NA | - | GH, thyrotropic  deficiency, PRL↑ | - | TSS | Grey-white, fish-meat like | | P53-, CD117+, PLAP+, OCT3/4+,  Ki-67: 40% | NA | | NA |
| 20 | | 8.9/M | GCT | 2 | - | NA | Binocular VA panhypo impairment, bitemporal VF defects | Thyrotropic, gonadotropic deficiency, PRL↑ | CSF: HCG+ | TSS | Grey-white, fish-meat like, soft | | HCG-, CD117+, PLAP+, OCT3/4+,  Ki-67: 70% | 8.7 | | Tumor partial resection, 3 IEP chemotherapy, radiotherapy, HRT(D+C+T)，no height increase or pubertal development |
| 21 | | 9.5/F | GCT | 8 | Growth retardation | BA:7-8  Ht:128.5(3-10%) | - | GH deficiency | - | TSS | Grey-white, fish-meat like | | β-HCG-, CD117+, PLAP+, OCT3/4+,  Ki-67: 70% | 10.1 | | Tumor partial resection, 4 EP chemotherapy, radiotherapy, HRT(C)，no height increase or pubertal development |
| 22 | | 6.4/F | GCT | 12 | Growth retardation | Ht:109(<3%) | - | GH, thyrotropic,  corticotropic deficiency, PRL↑ | Blood: HCG+  CSF: HCG+ | TSS | Grey-white, rubbery, fish-meat like | | HCG-, CD117+, PLAP+, OCT3/4+, S100+  Ki-67: 80% | 19.8 | | Tumor partial resection, 4 EP chemotherapy, radiotherapy, HRT(C)，no height increase or pubertal development |
| 23 | | 10.8/F | GCT | 36 | - | BA:10-11  Ht:138(3-10%) | - | GH deficiency | CSF: HCG+ | TSS | Grey-white, rubbery, fish-meat like | | P53-, CD117+, PLAP+  Ki-67: 40% | 73.0 | | Tumor partial resection, 4 EP chemotherapy, radiotherapy, HRT(D+C+T)，height increase, delayed puberty |
| 24 | | 7.7/F | GCT | 12 | Inappetence, growth retardation | BA:5-6  Ht:112(<3%) | - | GH, thyrotropic,  corticotropic deficiency, PRL↑ | - | TSS | Grey-white, soft | | CD117+, PLAP+ | 28.4 | | Tumor partial resection, 3 IEP chemotherapy, radiotherapy, HRT(D+T)，height increase, delayed puberty |
| 25 | | 11.2/F | GCT | 24 | Headache, hypodynamia, inappetence | NA | Binocular VA panhypo impairment, | GH, thyrotropic deficiency, PRL↑ | Blood: HCG+  CSF: HCG+ | TSS | Grey-white, rubbery, fish-meat like | | HCG+, CD117+, PLAP+  Ki-67: 40% | 18.1 | | Tumor partial resection, radiotherapy, HRT(D+C)，no height increase or pubertal development |
| 26 | | 11.1/M | GCT | 36 | Nausea and vomiting, growth retardation | BA:10  Ht:112(<3%) | - | Panhypo, PRL↑ | CSF: HCG+ | TSS | Grey-white, fish-meat like | | CD117+, PLAP- | 69.2 | | Tumor partial resection, radiotherapy, HRT(D+C+T+A)， height increase and normal pubertal development |
| 27 | | 5.8/F | GCT | 10 | - | Ht:110.6(3-10%) | - | GH, corticotropic deficiency | - | TSS | Grey-white, rubbery, fish-meat like | | P53+, CD117+, PLAP+  Ki-67: 60% | 7.1 | | Tumor partial resection, 4 EP chemotherapy, radiotherapy, HRT(D+C)，no height increase or pubertal development |
| 28 | | 9.5/F | GCT | 16 | Headache, inappetence, fever | Ht:131(10-25%) | Binocular VA panhypo impairment | GH, thyrotropic,  corticotropic deficiency, PRL↑ | - | TSS | Grey-white, rubbery, fish-meat like | | CD117+, PLAP+, OCT3/4+,  Ki-67: 30% | 14.6 | | Tumor partial resection, 4 EP chemotherapy, radiotherapy, HRT(D+C+T)，no height increase or pubertal development |
| 29 | | 8.8/F | GCT | 13 | Nausea and vomiting, fever, growth retardation | BA:5-6  Ht:122.5(3-10%) | - | GH, thyrotropic,  corticotropic deficiency, PRL↑ | Blood: HCG+  CSF: HCG+ | TSS | Grey-white, soft, fish-meat like | | P53±, β-HCG-, CD117+, PLAP-, OCT3/4+,  Ki-67: 60% | 34.1 | | Tumor partial resection, 3 IEP chemotherapy, radiotherapy, HRT(D+C+T)，height increase, delayed puberty |
| 30 | | 13.1/F | GCT | 36 | Headache, nausea and vomiting | Ht:140(<3%) | Binocular VA panhypo impairment, bitemporal VF defects | GH, thyrotropic,  corticotropic deficiency, electrolyte disturbance | - | TSS | Grey-white, little rubbery, fish-meat like | | HCG-, CD117+, PLAP+, OCT3/4+,  Ki-67: 40% | 34.9 | | Tumor partial resection, 3 IEP therapy, radiotherapy, HRT(D+C+G+T), height increase, delayed puberty，VF recovered |
| 31 | | 7.8/M | GCT | 16 | Growth retardation, penis enlargement | BA:6  Ht:116(<3%) | - | GH, thyrotropic deficiency, PRL↑, electrolyte disturbance | Blood: HCG+ | TSS | Grey-white, rubbery, fish-meat like | | CD117+, PLAP+ | NA | | NA |
| 32 | | 8.5/F | GCT | 16 | Nausea and vomiting, inappetence, growth retardation | BA：6-7  Ht:122(3-10%) | - | - | Blood: HCG+ | TSS | Grey-white, rubbery, fish-meat like | | P53-, HCG+, CD117+, PLAP+, OCT3/4+,  Ki-67: 30% | 33.3 | | Tumor partial resection, 4 EP chemotherapy, radiotherapy, HRT(D+C+T), height increase, delayed puberty |
| 33 | | 8.8/F | GCT | 55 | Headache, hypodynamia, inappetence | Ht137(50-75%) | Bitemporal VF defects, diplopia | GH, thyrotropic,  corticotropic deficiency, electrolyte disturbance | Blood: HCG+  CSF: HCG+ | craniotomy | Flesh pink, soft | | P53-, HCG±, β-HCG+, CD117+, PLAP+, OCT3/4+, CD30+,  Ki-67: 70% | 75.2 | | Tumor subtotal resection, 4 EP chemotherapy, radiotherapy, HRT(D+C+G+T), height increase, delayed puberty |
| 34 | | 8.3/F | GCT | 19 | Headache, hypodynamia, inappetence | Ht:135(50-75%) | - | GH, corticotropic deficiency , PRL↑ | CSF: HCG+ | TSS | Grey-white, rubbery, fish-meat like | | P53+, HCG-, β-HCG-, CD117+, PLAP+  Ki-67: 60% | 17.8 | | Tumor partial resection, 3 EP chemotherapy, radiotherapy, HRT(D+C+G+T), height increase, delayed puberty |
| 35 | | 6.6/M | GCT | 9 | Headache, growth retardation | BA:4  Ht:124.5(75-90%) | - | GH deficiency, PRL↑, electrolyte disturbance | CSF: HCG+ | TSS | Grey-white, rubbery, fish-meat like | | CD117+, PLAP+ | 40.0 | | Tumor partial resection, 4 EP chemotherapy, radiotherapy, HRT(D+C), no height increase or pubertal development |
| 36 | | 7.4/F | GCT | 48 | Headache, nausea and vomiting, inappetence, growth retardation | Ht:110(<3%) | - | GH, thyrotropic deficiency, PRL↑, electrolyte disturbance | - | Crani-otomy | Grey-pink | | CD117+, PLAP- | 6.0 | | Radiotherapy and 4 VMPP chemotherapy, tumor total resection, died 6 months after the surgery due to hypothalamic dysfunction |
| 37 | | 9.1/F | GCT | 9 | Hypodynamia | Ht:133(25-50%) | - | GH, thyrotropic, corticotropic deficiency | CSF: HCG+ | TSS | Grey-white, little soft | | CD117+, PLAP+ | 32.4 | | Tumor partial resection, 4 EP chemotherapy, radiotherapy, HRT(D+C+T), no height increase or pubertal development |
| 38 | | 8.3/F | GCT | 1 | Headache, nausea and vomiting, hypodynamia, inappetence | BA:8  Ht:127(25-50%) | - | Thyrotropic deficiency, electrolyte disturbance | - | TSS | Dark grey, rubbery, fish-meat like | | CD117+, PLAP+ | 46.3 | | Tumor partial resection, 4 EP chemotherapy, radiotherapy, HRT(D+C+G+T), height increase and normal pubertal development |
| 39 | | 12.3/F | GCT | 36 | Headache, hypodynamia, inappetence, growth retardation | Ht:139(<3%) | Bitemporal VF defects | GH, thyrotropic deficiency, PRL↑ | Blood: HCG+  CSF: HCG+ | TSS | Grey-white, rubbery, little soft | | CD117+, PLAP+, OCT3/4+ | 47.3 | | Tumor partial resection, 3 IEP chemotherapy, radiotherapy, HRT(D+C+T), no height increase or pubertal development, VF recovered |
| 40 | | 7.8/M | GCT | 1 | Headache, penis enlargement | Ht:136(75-90%) | - | T↑, PRL↑, electrolyte disturbance | Blood: AFP+&HCG+  CSF: HCG+ | Crani-otomy | Grey-pink, jellylike | | P53+, β-HCG±, CD117+, PLAP+, OCT3/4+, CD30+  Ki-67: 80% | 21.7 | | Tumor subtotal resection, 4 EP chemotherapy, radiotherapy, HRT(D+C+T), height increase and normal pubertal development |
| 41 | | 9.6/M | GCT | 24 | Headache, inappetence, growth retardation | Ht:122(<3%) | - | GH deficiency, PRL↑, electrolyte disturbance | CSF: HCG+ | Crani-otomy | Grey-pink | | P53±, β-HCG±, CD117+, PLAP+  Ki-67: 50% | 81.6 | | Tumor subtotal resection, 4 EP chemotherapy, radiotherapy, HRT(D+C+G), height increase but no pubertal development |
| 42 | | 14/F | LCH | 5 | Headache, amenorrhoea | Ht;160(50-75%) | - | - | - | Bone biopsy | | NA | CD1a+, S100+,  Ki-67: 3% | 53.8 | | 2 MA chemotherapy, HRT(D), height increase and normal pubertal development |
| 43 | | 14/F | LCH | 18 | - | BA:14-16  Ht:160(50-75%) | - | GH deficiency, electrolyte disturbance | - | Bone biopsy | | NA | CD1a+, S100+, | 65.5 | | 3 MA + 2 COEP chemotherapy, HRT(D), height increase and normal pubertal development |
| 44 | | 12.3/F | LCH | 35 | - | Ht:146(10-25%) | - | GH deficiency | - | TSS | Grey-white, rubbery | | CD1a+, S100+, | 45.7 | | 6 months after 52 weeks of VMP chemotherapy, LCH recurrence in CNS, then received radiotherapy, HRT(D+C), height increase but no pubertal development |
| 45 | | 13.3/M | LCH | 4 | Headache, nausea and vomiting | Ht:160(50-75%) | - | Panhypo, electrolyte disturbance | - | TSS | Grey-white, fish-meat like | | CD1a+, S100+, CD117-, PLAP- | 34.8 | | Radiotherapy, HRT(D+C+T), height increase but no pubertal development |
| 46 | | 14/M | LCH | 2 | Headache | BA: 18  Ht:169.5(50-75%) | - | PRL↑ | - | TSS | Grey-white, rubbery | | P53-  CD1a+, S100+, langerin+  Ki-67: 8% | 36.3 | | 6 MA chemotherapy， HRT(D), height increase and normal pubertal development, VF bitemporal defects |
| 47 | | 11.6/M | LCH | 10 | Headache | Ht:151(50-75%) | - | Gonadotropic deficiency | - | Bone biopsy | | NA | CD1a+, S100+, | 5.5 | | 14 V chemotherapy, HRT (D), height increase but no pubertal development |
| 48 | | 12.5M | LCH | 4 | - | Ht:164(75-90%) | Left eye temporal VF defect | Gonadotropic deficiency | NA | Bone biopsy | | NA | CD1a+, S100+, | NA | | NA |
| 49 | | 9.25/F | LCH | 2 | Nausea and vomiting, inappetence, growth retardation | BA: 7-8  Ht:115(<3%) | - | GH, thyrotropic, gonadotropic deficiency, PRL↑ | - | Cranio-tomy | | NA | CD1a+, S100+, P53-, CD117±  Ki-67: 1% | 46.1 | | Radiotherapy, HRT(D+T), no height increase or pubertal development |
| 50 | | 3.58/F | LCH | 108 | Headache, nausea and vomiting, inappetence, growth retardation | Ht: 92(<3%) | - | GH deficiency, PRL↑ | - | Bone biopsy | | NA | CD1a+, S100+,  Ki-67: 15% | 50.1 | | 9 VM + 9 VP chemotherapy, HRT(D+T), no height increase or pubertal development |
| 51 | | 7.33/M | LCH | 23 | Growth retardation | BA: 2  Ht:107(<3%) | - | GH, gonadotropic deficiency, electrolyte disturbance | - | Bone biopsy | | NA | CD1a+, S100+,  Ki-67: 1% | 52.4 | | 5 VP chemotherapy, HRT(D), no height increase or pubertal development |
| 52 | | 10.5/M | CP | 3 | - | Ht:140(25-50%) | - | Corticotropic, gonadotropic deficiency, PRL↑ | NA | TSS | Oil-like crystal and solid tumor | | Remote hemorrhage, cholesterol crystals, foam cells, multinucleated giant cell response | | 20.7 | Tumor total resection, HRT(D+C+T), height increase, delayed puberty |
| 53 | | 13.1/F | CP | 5 | - | Ht: 151(10-25%) | - | - | - | TSS | Oil-like crystal and soft tumor | | Necrosis tissue, cholesterol crystals, inflammatory cells | | 42.4 | Tumor total resection, HRT(D), height increase and normal pubertal development |
| 54 | | 8/F | CP | 60 | Growth retardation | Ht:110(<3%) | Left eye VA deficient, right eye blindness | GH，thyrotropic, corticotropic deficiency | NA | TSS | Oil-like cystic fluid, yellow-white tumor | | Necrosis tissue, cholesterol crystals | 19.3 | | Tumor total resection, no height increase or pubertal development, no improvement in ophthalmic examination |
| 55 | | 14/F | CP | 72 | Growth retardation | Ht: 142(<3%) | - | GH, thyrotropic deficiency | - | TSS | Grey-white, soft | | Ki-67: 2% | 47.6 | | Tumor total resection, HRT(D+T), no height increase or pubertal development |

* Tumor marker：+ indicated value higher than normal range, - indicated value between normal range.

* Abbreviation:

TSS: transsphenoidal surgery

GCT: germ cell tumor

LCH: Langerhans cell histiocytosis

CP: Craniopharyngioma

Ht: height

BA: bone age

VA: visual acuity

VF: visual field

SSC：secondary sex characteristics

EP: VP-16+Cis-platinum

IEP: Isophosphoramide (I)+Etoposide (E)+Cis-platinum (P)

VMPP: vincristine (V)+methotrexate (M)+pingyangmycin (P)+Cis-platinum (P)

MA: mitoxantrone(M) + Ara-c(A)

VMP: Vindesine(V) + Methotrexate(M) + Prednisone(P)

VM: Vindesine(V) + Methotrexate(M)

VP: vinbalstin (V) + Prednisone(P)

D: DDAVP(Minirin)

C: corticosteroid (Hydrocortisone、prednison)

G: growth hormone(Jintropin)

T: Thyroxine (Euthyrox or Letrox)

E: Estrogen

A: Androgen (Testosterone)

NA: not accessible
